# Association between blood urea nitrogen and the prevalence of Hashimoto's thyroiditis in adults with type 2 diabetes mellitus: a cross-sectional study

**DOI:** 10.3389/fnut.2026.1769524

**Published:** 2026-02-20

**Authors:** Mengni He, Zhenjun Yu, Shaojie Duan, Ping Feng, Qidong Zheng, Shuijiao Liu, Yishan Yin, Mengdie Chen

**Affiliations:** 1 Department of Gastroenterology, Taizhou Central Hospital (Taizhou University Hospital), Taizhou, Zhejiang, China; 2 Department of Geriatrics, Taizhou Central Hospital (Taizhou University Hospital), Taizhou, Zhejiang, China; 3 Department of Endocrinology, Taizhou Central Hospital (Taizhou University Hospital), Taizhou, Zhejiang, China; 4 Department of Internal Medicine, Yuhuan Second People's Hospital, Yuhuan, Zhejiang, China; 5 Department of Orthopedics, The Armed Police Forces Hospital of Shandong, Jinan, China

**Keywords:** blood urea nitrogen, cross-sectional study, Hashimoto's thyroiditis, nonlinear relationship, type 2 diabetes mellitus

## Abstract

**Background:**

Blood urea nitrogen (BUN), a known marker of renal function and protein catabolic status, is essential for inflammatory and metabolic dysregulation. There is currently little information available on the connection between BUN and Hashimoto's thyroiditis (HT) in individuals with type 2 diabetes mellitus (T2DM). The purpose of this study was to look into the relationship between HT and BUN in T2DM patients.

**Methods:**

Two thousand fifty four adult T2DM patients from two hospitals were included in this cross-sectional analysis of data from the National Metabolic Management Center (MMC) cohort. The independent relationship between BUN and HT was evaluated using multivariable logistic regression models. Restricted cubic spline (RCS) regression was utilized to examine potential nonlinear correlations. The consistency of correlations across strata of sex, age, body mass index (BMI), and study center was assessed by subgroup analysis.

**Results:**

HT was present in 20.2% of cases (414/2054). Each 1 mmol/L rise in BUN was linked to a 7% greater chance of HT after adjusting for age, sex, education, duration of diabetes, BMI, HbA1c, smoking, drinking, hypertension, and hyperlipidemia (OR = 1.07, 95% CI: 1.01–1.13, *P* = 0.027). BUN and HT were shown to have a nonlinear relationship with an inflection point at 5.299 mmol/L. A 52.1% increase in prevalence of HT was linked to every 1 mmol/L increase when BUN was ≤5.299 mmol/L (OR = 1.521, 95% CI: 1.196–1.934, *P* < 0.001). All subgroups had consistent positive connections, according to subgroup analysis (all interaction *P* > 0.05).

**Conclusion:**

Higher levels of BUN, particularly within a lower-to-moderate range, are independently associated with an increased prevalence of HT in patients with T2DM. These results imply that BUN, a commonly accessible metric, may be useful in identifying T2DM patients who are more likely to have concurrent autoimmune thyroiditis.

## 1 Introduction

Chronic hyperglycemia and insulin resistance are hallmarks of type 2 diabetes mellitus (T2DM), a common metabolic condition that can have serious consequences such as neuropathy, nephropathy, and cardiovascular disease (1–3). It is a significant worldwide public health issue. The prevalence is estimated to be 537 million individuals globally, and by 2030, it is expected to increase to 643 million (4). In China, where the frequency among adults has reached 11.2% (5), the issue is especially worrisome since it greatly increases the country's burden of non-communicable illnesses and healthcare expenses (6).

Blood urea nitrogen (BUN), a crucial indication of renal function and protein metabolism, has drawn interest in the hunt for early biomarkers and modifiable risk factors for diabetes-associated comorbidities. Elevated BUN may be a sign of low-grade inflammation, metabolic stress, and altered nitrogen balance, which may have an impact on immune modulation and autoimmunity, according to emerging experimental findings (7–9). In vulnerable groups like those with T2DM, this suggests a biological connection between BUN and the risk of autoimmune diseases like Hashimoto's thyroiditis (HT).

Despite the biological plausibility, there is still little and conflicting epidemiological evidence linking BUN to HT. Thyroid autoimmunity and renal function markers may be related, according to a few general population studies (10, 11). However, small sample sizes, a lack of thorough correction for metabolic confounders, and a lack of attention to possible nonlinear correlations frequently limit the scope of current research. Crucially, there is a dearth of evidence explicitly analyzing this link in individuals with T2DM, a population at high risk for both autoimmune thyroid disease and renal failure.

Therefore, to address these gaps, we conducted a cross-sectional analysis utilizing data from the National Metabolic Management Center (MMC) cohort, and aimed to investigate the association between BUN levels and the prevalence of HT in patients with T2DM.

## 2 Materials and methods

### 2.1 Study participants

**Figure 1:**
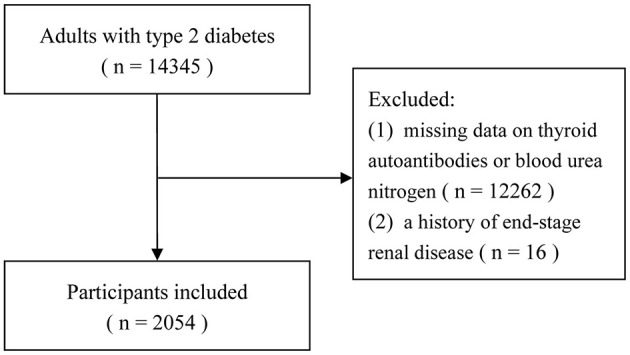
The flow chart of the study.

Data from the MMC cohort at Taizhou Central Hospital (Taizhou University Hospital) and Yuhuan Second People's Hospital from September 2017 to February 2025 were used in this cross-sectional analysis. Initially, 14,345 people with T2DM were screened. Lacking thyroid autoantibody or BUN values or a history of end-stage renal disease were the two exclusion criteria. Participants were excluded if they met any of the following criteria: (1) missing data for BUN or thyroid autoantibodies (TG-Ab and/or TPO-Ab), which were essential for exposure assessment and covariate adjustment; (2) a documented history of end-stage renal disease (ESRD), given that severe renal impairment independently alters BUN metabolism and could confound the studied association. In the end, 2,054 individuals were included in the study (the flow chart is shown in Figure 1). The Clinical Research Ethics Committees of both participating hospitals accepted the study procedure. Prior to recruitment, all participants provided written informed permission, and the study was carried out in compliance with the Declaration of Helsinki's guiding principles.

### 2.2 Data collection

Each person underwent a thorough clinical assessment that included behavioral aspects, biological indicators, and sociodemographic traits. Age, sex, education level, duration of diabetes, history of hypertension and hyperlipidemia, smoking and drinking status, height, weight, systolic blood pressure (SBP), diastolic blood pressure (DBP), fasting blood glucose (FBG), fasting C-peptide (FCp), glycated hemoglobin (HbA1c), total cholesterol (TC), triglycerides (TG), high-density lipoprotein cholesterol (HDL-C), uric acid (UA), serum creatinine (Scr), BUN, free triiodothyronine (FT3), free thyroxine (FT4), thyroid-stimulating hormone (TSH), anti-thyroglobulin antibody (TG-Ab), and anti-thyroid peroxidase antibody (TPO-Ab). Blood samples were collected after an overnight fast of at least 8 h and processed using standardized laboratory protocols. FBG was measured using the hexokinase method; FCp was quantified by chemiluminescent microparticle immunoassay (CMIA); and HbA1c was analyzed by high-performance liquid chromatography (HPLC) calibrated to National Glycohemoglobin Standardization Program (NGSP) standards. For renal function assessment, BUN was determined using the enzymatic continuous monitoring method (urease-glutamate dehydrogenase), Scr was measured by the sarcosine oxidase method, and UA was assayed using the uricase-peroxidase (Uricase-PAP) method. Lipid profiles were assessed as follows: TG by the glycerol kinase-glycerol phosphate oxidase-peroxidase method (GK-GPO-PAP), TC by the cholesterol oxidase-peroxidase method (CHOD-PAP), and both HDL-C and LDL-C by direct homogeneous endpoint methods. Thyroid function parameters were measured using distinct immunoassay methodologies: FT3 and FT4 by competitive methods, TSH by a two-site sandwich method, and both TG-Ab and TPO-Ab by competitive chemiluminescent microparticle immunoassays. The body mass index (BMI) was computed by weight (kg)/height (m^2^). Based on Scr levels, the Chronic Kidney Disease Epidemiology Collaboration equation was used to determine the estimated glomerular filtration rate (eGFR) (12).

### 2.3 Definition of Hashimoto's thyroiditis

Thyroid autoantibodies in serum were used to define HT (13). Antibody positivity was determined as TPOAb > 34 IU/ml and/or TgAb > 115 IU/ml based on the manufacturer's recommended limit. The HT-positive group consisted of individuals who satisfied the positivity criteria for either antibody.

### 2.4 Statistical analyses

While continuous variables were shown as mean (standard deviation, SD) or median (interquartile range, IQR), depending on their distribution, categorical variables were reported as proportions (%). One-way ANOVA for normally distributed data, Kruskal-Wallis tests for skewed data, or chi-square testing for categorical data were used to evaluate group differences. Multiple imputation was used to manage missing data; Supplementary Table 1 contains information on covariate missingness. The relationship between BUN and HT was assessed using logistic regression models, which produced odds ratios (OR) with 95% confidence intervals (95% CI). BUN was classified into tertiles and analyzed as a continuous variable. A crude model with no adjustments was created; Model 1 was adjusted for age and sex; Model 2 was further adjusted for education level, diabetes duration, body mass index, and HbA1c; and Model 3 was fully adjusted for age, sex, education level, diabetes duration, BMI, HbA1c, smoking, drinking, hypertension, and hyperlipidemia. Based on Model 3, restricted cubic spline (RCS) regression was used to evaluate the linearity of the BUN–HT association. Likelihood-ratio tests and bootstrap resampling were performed to discover possible inflection points in a two-piecewise logistic regression model with smoothing if non-linearity was apparent. Using multivariate logistic regression, potential impact modification was investigated among subgroups defined by sex, age (< 60 vs. ≥60 years), BMI (< 24 vs. ≥24 kg/m^2^), and study center. Likelihood-ratio tests were used to examine interactions between subgroups and BUN. Only full instances were used in sensitivity studies to assess any potential bias caused by imputation. R 4.2.2 R Statistical Software (Version 4.2.2, http://www.R-project.org, The R Foundation) and Free Statistics software (Version 2.3.1, Beijing, China, http://www.clinicalscientists.cn/freestatistics) were used for all analyses, and the threshold for statistical significance was set at a two-sided *P* < 0.05.

## 3 Results

### 3.1 Baseline characteristics of the study population

The final analysis of this cross-sectional study includes 2,054 individuals. Table 1 displays the study population's baseline characteristics. 414 people, or 20.2% of the study population, were diagnosed with HT out of the entire cohort. BUN tertiles were used to stratify the participants into three groups: T1 (BUN ≤ 4.7 mmol/L), T2 (BUN > 4.7 to ≤ 6.0 mmol/L), and T3 (BUN > 6.0 mmol/L). Several baseline factors showed statistically significant differences (*P* < 0.05) in inter-group comparisons. Participants showed a higher percentage of males, older age, longer duration of diabetes, and a higher prevalence of hypertension (*P* < 0.001) as BUN tertiles increased. Renal function indicators also changed at the same time: eGFR dropped and Scr levels rose dramatically (*P* < 0.001). Additionally, there were differences in HT prevalence between the tertiles (T1: 15.2%; T2: 23.3%; T3: 21.9%, *P* < 0.001). There were no statistically significant differences between the BUN groups in other metabolic and thyroid function indicators, including FBG, lipid profiles, FT3, and TSH.

**Table 1:** Baseline characteristics of the study participants.

**Variables**	**Total (*n* = 2,054)**	**BUN, mmol/L**			***P*-value**
		**T1 (** ≤ **4.7;** ***n*** = **683)**	**T2 (**>**4.7**, ≤ **6;** ***n*** = **681)**	**T3 (**>**6;** ***n*** = **690)**	
Male, *n* (%)	1,300 (63.3)	406 (59.4)	419 (61.5)	475 (68.8)	< 0.001
Age, years	54.5 ± 12.8	49.6 ± 12.7	54.5 ± 12.4	59.2 ± 11.6	< 0.001
High school education and above, *n* (%)	409 (19.9)	197 (28.8)	123 (18.1)	89 (12.9)	< 0.001
DBP, mmHg	77.1 ± 11.0	78.2 ± 10.2	77.5 ± 11.2	75.7 ± 11.3	< 0.001
SBP, mmHg	130.8 ± 16.9	129.7 ± 16.2	130.8 ± 16.5	131.9 ± 18.0	0.071
BMI, kg/m^2^	25.4 ± 3.8	25.8 ± 4.1	25.3 ± 3.7	25.2 ± 3.4	0.004
Duration of diabetes, years	3.0 (0.1, 9.8)	1.2 (0.0, 6.8)	3.0 (0.1, 9.1)	4.9 (0.8, 12.1)	< 0.001
Hypertension, *n* (%)	1,105 (53.8)	314 (46)	357 (52.4)	434 (62.9)	< 0.001
Hyperlipidemia, *n* (%)	1,460 (71.1)	473 (69.3)	495 (72.7)	492 (71.3)	0.371
Smoking, *n* (%)	509 (24.8)	165 (24.2)	169 (24.8)	175 (25.4)	0.875
Drinking, *n* (%)	298 (14.5)	94 (13.8)	101 (14.8)	103 (14.9)	0.794
FBG, mmol/L	9.2 ± 3.7	9.4 ± 3.7	9.2 ± 3.6	9.0 ± 3.6	0.126
FCp, ng/ml	2.3 ± 1.4	2.1 ± 1.5	2.2 ± 1.2	2.4 ± 1.6	< 0.001
HbA1c, %	9.1 ± 2.5	9.6 ± 2.6	8.8 ± 2.3	8.8 ± 2.4	< 0.001
BUN, mmol/L	5.6 ± 2.0	3.8 ± 0.7	5.3 ± 0.4	7.6 ± 2.0	< 0.001
Scr, μmol/L	67.9 ± 27.1	58.6 ± 15.0	63.6 ± 16.0	81.3 ± 37.8	< 0.001
e-GFR, ml/min per 1.73 m^2^	107.1 ± 33.3	121.8 ± 31.6	109.1 ± 28.0	90.7 ± 32.6	< 0.001
UA, μmol/L	334.0 ± 100.1	314.9 ± 95.2	326.8 ± 95.6	359.8 ± 104.0	< 0.001
TG, mmol/L	1.6 (1.1, 2.5)	1.7 (1.2, 2.5)	1.7 (1.2, 2.5)	1.6 (1.1, 2.4)	0.155
TC, mmol/L	5.1 ± 1.3	5.0 ± 1.3	5.1 ± 1.3	5.2 ± 1.4	0.176
HDL-C, mmol/L	1.1 ± 0.3	1.1 ± 0.3	1.1 ± 0.3	1.1 ± 0.3	0.076
LDL-C, mmol/L	3.0 ± 1.0	3.0 ± 1.0	3.0 ± 1.0	3.0 ± 1.0	0.899
FT3, pg/ml	2.9 ± 1.1	2.9 ± 0.8	3.0 ± 1.4	2.9 ± 1.2	0.19
FT4, ng/dl	1.2 ± 0.4	1.2 ± 0.4	1.2 ± 0.4	1.2 ± 0.4	0.298
TSH, μIU/ml	1.5 (1.0, 2.1)	1.5 (1.0, 2.2)	1.5 (1.0, 2.1)	1.5 (1.0, 2.1)	0.726
TPOAb, IU/ml	9.0 (0.5, 14.0)	9.0 (0.6, 13.3)	9.0 (0.5, 15.0)	2.1 (0.4, 13.0)	< 0.001
TGAb, IU/ml	11.0 (0.9, 16.0)	13.0 (1.0, 16.0)	11.0 (0.9, 17.0)	3.1 (0.9, 16.0)	0.03
HT, *n* (%)	414 (20.2)	104 (15.2)	159 (23.3)	151 (21.9)	< 0.001

T, tertile; SBP, systolic blood pressure; DBP, diastolic blood pressure; BMI, body mass index; FBG, fasting blood glucose; FCp, fasting serum C peptide; HbA1c, glycated hemoglobin; UA, uric acid; BUN, blood urea nitrogen; Scr, serum creatinine; eGFR, estimated glomerular filtration rate; TG, triglycerides; TC, total cholesterol; HDL-C, high-density lipoprotein cholesterol; LDL-C, low-density lipoprotein cholesterol; TPO-Ab, anti-thyroid peroxidase antibody; TG-Ab, anti-thyroglobulin antibody; HT, Hashimoto's thyroiditis.

### 3.2 Association between BUN and HT in T2DM adults

Supplementary Table 2 displays the findings of the univariate logistic regression study. Elevated BUN was substantially linked to a higher incidence of HT among all the factors examined (OR = 1.06, 95% CI: 1.01–1.12, *P* = 0.020). BUN's relationship with HT became clearer when it was examined as a categorical variable (stratified by tertiles). The middle and highest BUN tertiles were linked to a considerably higher prevalence of HT (*P* < 0.05) when the lowest BUN tertile was used as the reference. A higher incidence of HT was linked to lower educational level, DBP, and eGFR (*P* < 0.05). In this univariate study, additional factors such as age, sex, blood pressure, diabetes duration, lipid profiles, and other thyroid function indicators did not reach statistical significance.

Multivariate logistic regression analysis assessed the independent relationship between BUN and HT following stepwise correction for possible confounders, as indicated in Table 2. Both when BUN was examined as a continuous variable and as a categorical variable (tertiles), the positive correlation between BUN and HT persisted across all correction models. When analyzed as a continuous variable, each 1 mmol/L increase in BUN was consistently associated with approximately a 7% increase in prevalence of HT after adjusting for age and sex (Model 1), with further adjustments for education level, diabetes duration, BMI, and HbA1c (Model 2, OR = 1.06, 95% CI: 1.01–1.13, *P* = 0.030), and with full adjustment for smoking, drinking, hypertension, and hyperlipidemia (Model 3, OR = 1.07, 95% CI: 1.01–1.13, *P* = 0.031). In Model 3, both the middle (T2) and highest (T3) BUN tertiles were significantly associated with higher odds of HT, corresponding to ORs of 1.67 (95% CI: 1.26–2.22, *P* < 0.001) and 1.56 (95% CI: 1.16–2.11, *P* = 0.003), respectively, when compared with the lowest tertile (T1).

**Table 2:** Association between BUN and HT in patients with T2DM based on multivariate logistic regression analysis.

**Variable**	**Crude**		**Model 1**		**Model 2**		**Model 3**	
	**OR (95% CI)**	***P*** **-value**	**OR (95% CI)**	***P*** **-value**	**OR (95% CI)**	***P*** **-value**	**OR (95% CI)**	***P*** **-value**
BUN, mmol/L	1.06 (1.01–1.12)	0.020	1.07 (1.01–1.13)	0.016	1.06 (1.01–1.13)	0.030	1.06 (1.01–1.13)	0.031
**BUN tertiles**								
T1	1 (Ref)		1 (Ref)		1 (Ref)		1 (Ref)	
T2	1.7 (1.29–2.23)	< 0.001	1.73 (1.31–2.28)	< 0.001	1.65 (1.25–2.19)	< 0.001	1.67 (1.26–2.22)	< 0.001
T3	1.56 (1.18–2.06)	0.002	1.63 (1.21–2.18)	0.001	1.56 (1.16–2.09)	0.004	1.57 (1.16–2.11)	0.003

Crude: no adjustment; Model 1: adjusted for sex and age; Model 2: adjusted for model 1 + education level, duration of diabetes, BMI, and HbA1c; Model 3: adjusted for model 2 + smoking, drinking, hypertension, hyperlipidemia.

**Figure 2:**
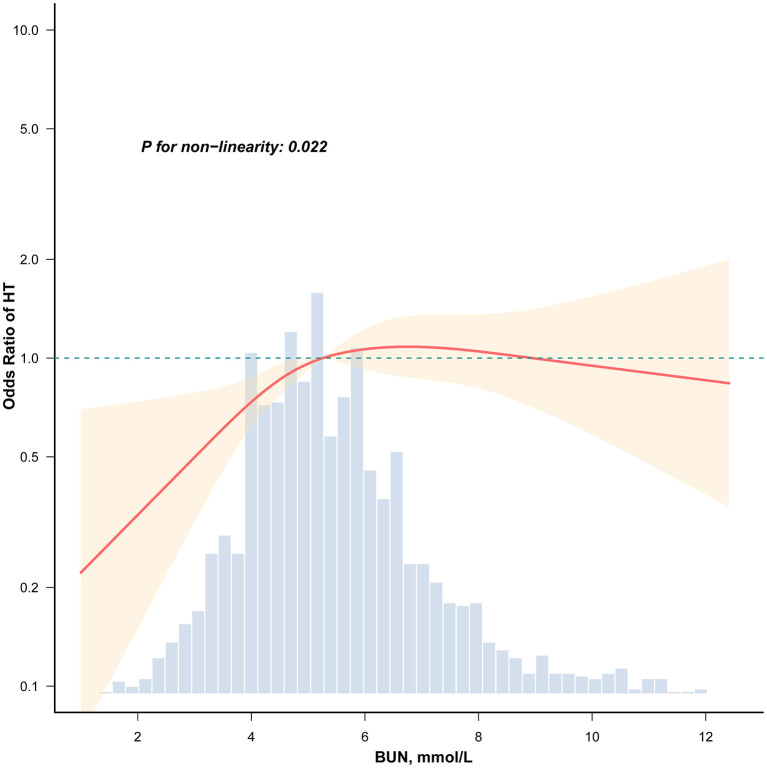
Association between BUN and HT among T2DM patients. Adjusted for age, sex, education level, duration of diabetes, BMI, HbA1c, smoking, drinking, hypertension, and hyperlipidemia. Only 99% of the data is shown.

The prevalence of HT and BUN levels had a strong nonlinear association, according to RCS analysis (Figure 2, *P* for nonlinearity = 0.022). The nonlinear association between BUN and HT was validated by threshold effect analysis (Table 3). 5.299 mmol/L was found to be a major tipping point. A dose-response association was evident on the left side of the inflection point (BUN ≤ 5.299 mmol/L), where a 1 mmol/L rise in BUN was linked to a 52.1% higher prevalence of HT (OR = 1.521, 95% CI: 1.196–1.934, *P* < 0.001). However, there was no statistically significant correlation between BUN and HT prevalence on the right side of the inflection point (BUN > 5.299 mmol/L; OR = 0.977, 95% CI: 0.868–1.099, *P* = 0.700).

**Table 3:** Threshold effect analysis of the relationship of BUN with HT.

**BUN, mmol/L**	**Adjusted model**	
	**OR (95% CI)**	***P*** **-value**
≤ 5.299	1.521 (1.196–1.934)	< 0.001
>5.299	0.977 (0.868–1.099)	0.700
Likelihood ratio test		0.001

OR, odds ratio; CI, confidence interval. Adjusted for age, sex, education level, duration of diabetes, BMI, HbA1c, smoking, drinking, hypertension, and hyperlipidemia. Only 99% of the data is displayed.

### 3.3 Subgroup analysis

**Figure 3:**
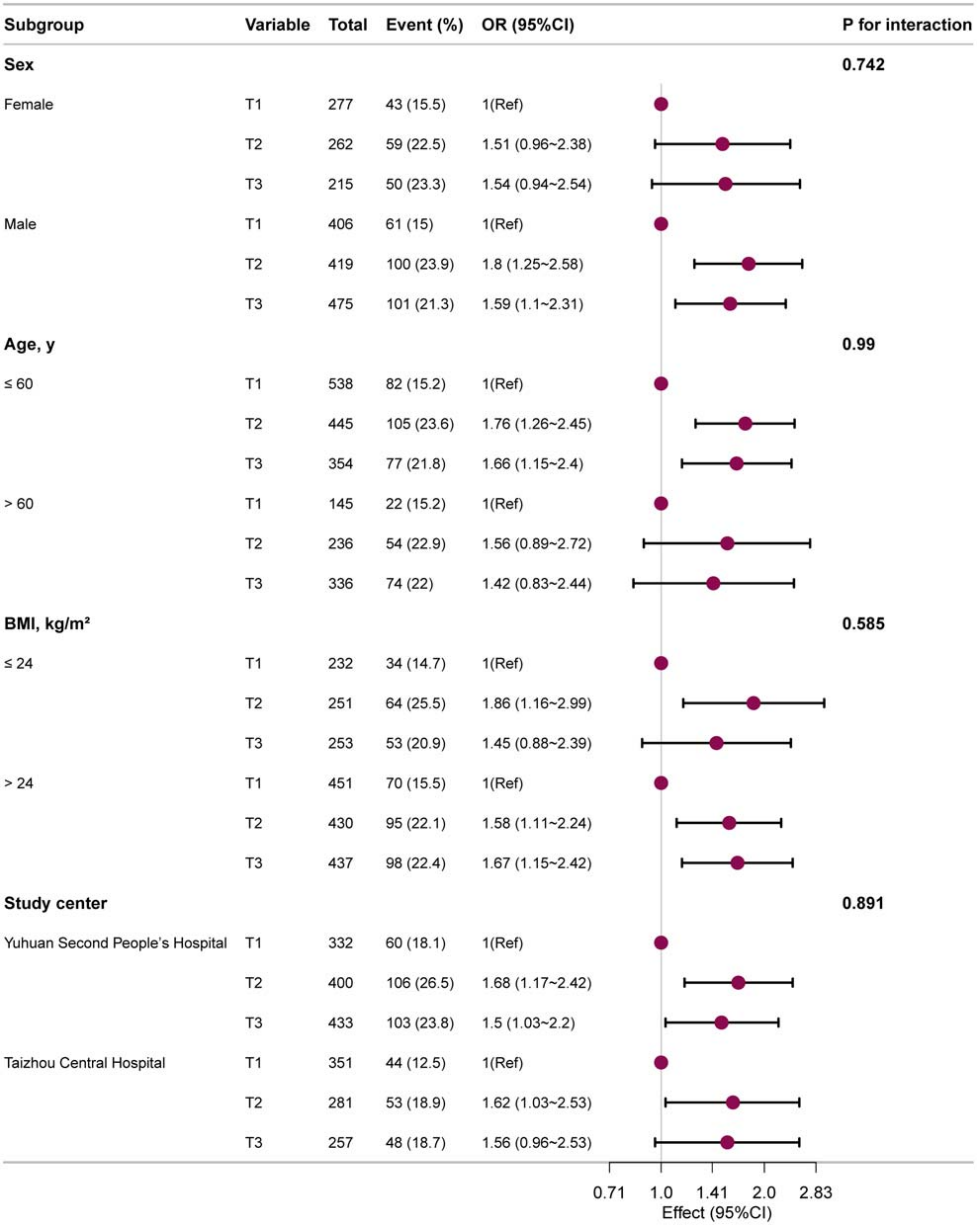
Subgroup analysis of the BUN and HT among T2DM patients. Each stratification factor, aside from the stratification component, was adjusted considering all other variables including age, sex, education level, diabetes duration, BMI, HbA1c, smoking, drinking, hypertension, and hyperlipidemia.

Subgroup studies were carried out to evaluate the generalizability of the relationship between BUN and HT. The results (Figure 3) consistently demonstrated a positive correlation between elevated BUN and increased HT prevalence across all categories after stratification by sex, age, BMI, and research center. In particular, higher BUN levels were linked to an increased prevalence of HT among each subgroup, with effects trending in the same direction. The strength of the link between BUN and HT was not substantially altered by sex, age, BMI, or research center, according to interaction analyses (all interaction *P*-values > 0.05).

### 3.4 Sensitivity analysis

To confirm that our primary findings were robust, sensitivity analyses were carried out. Both analyses limited to full cases and studies utilizing datasets with multiple imputation for missing data showed the same results (Supplementary Table 3).

## 4 Discussion

This cross-sectional study demonstrates that higher levels of BUN are independently associated with an increased prevalence of HT in patients with T2DM. Furthermore, we identified a significant nonlinear (threshold) relationship, with the association being particularly pronounced and showing a clear dose-response pattern when BUN levels are ≤ 5.299 mmol/L. The robustness of this association is supported by consistent findings across all examined subgroups stratified by sex, age, BMI, and study center.

BUN is a well-established and routinely measured biomarker of renal function and protein catabolic state (14). While traditionally considered a reflection of glomerular filtration rate and dietary protein intake, emerging epidemiological evidence has linked elevated BUN, even within the normal range, to systemic inflammation, oxidative stress, and adverse cardiovascular outcomes (15–17). Our findings align with a growing body of research suggesting a connection between metabolic-renal parameters and autoimmune thyroid diseases (18). For instance, studies have reported associations between impaired renal function, as indicated by reduced eGFR, and the presence of thyroid autoantibodies (19, 20). Notably, our study identifies BUN, an often-overlooked parameter distinct from eGFR in reflecting nitrogenous waste accumulation and catabolic state, as an independent predictor of HT in T2DM patients. This distinction is clinically relevant because BUN levels may rise earlier than eGFR declines in the trajectory of metabolic deterioration, potentially offering a more sensitive window for autoimmune risk stratification. Notably, to our knowledge, this is the first investigation to delineate a nonlinear threshold effect (inflection point 5.299 mmol/L), suggesting that the risk gradient is particularly steep within the lower-to-moderate BUN spectrum.

The observed association between BUN and HT may be explained through several interconnected pathways. First, elevated BUN may reflect or contribute to a state of low-grade systemic inflammation and immune dysregulation (16, 21–24). Urea at elevated concentrations has been shown in experimental models to promote oxidative stress and activate inflammatory pathways (25–27), which are key drivers in the pathogenesis of autoimmune diseases like HT. Second, BUN levels are intricately linked to protein metabolism and nitrogen balance. Dysregulated protein catabolism may alter the availability of amino acids crucial for immune cell function and tolerance, potentially facilitating autoimmune responses (27–29). Third, increased BUN often coexists with other metabolic disturbances common in T2DM (30), such as insulin resistance and hyperlipidemia, which themselves may create a pro-autoimmune milieu. Thus, BUN might serve as an integrative marker of metabolic stress that potentiates the risk of concurrent autoimmunity in susceptible individuals.

The observed association indicates that BUN levels, obtained from a simple and routine test, may serve as a practical screening indicator to help identify T2DM patients with a higher prevalence of concomitant HT. We emphasize that these findings establish correlation rather than causation, given the observational design and single time-point measurements. Nevertheless, this study offers distinct clinical utility: First, we delineate a nonlinear risk pattern concentrated within the lower-to-moderate BUN range ( ≤ 5.299 mmol/L), suggesting that even modest elevations within conventionally “normal” parameters may signal heightened HT susceptibility, enabling earlier risk stratification than reliance on overt renal dysfunction alone. Second, our findings generate the testable hypothesis that metabolic optimization to maintain BUN within an optimal range might influence thyroid autoimmunity trajectory, though this requires prospective interventional validation. Third, from a pragmatic standpoint, incorporating BUN assessment into routine T2DM management provides a cost-effective, universally available metric to prioritize thyroid autoantibody screening in high-risk subsets, supporting a more integrated metabolic-endocrine surveillance strategy.

Major strengths of this study include its relatively large sample size drawn from a standardized metabolic management cohort, comprehensive adjustment for a wide array of potential confounders including glycemic control and lipid profiles, and the use of restricted cubic spline analysis to robustly examine and characterize the nonlinear association. The consistency of results across subgroups and in sensitivity analyses further strengthens the findings.

However, several limitations must be acknowledged. First, the cross-sectional design precludes definitive conclusions regarding causality or the direction of the association. Second, BUN was measured at a single time point and can be influenced by short-term factors like hydration status, dietary protein intake, and acute illness, which were not fully accounted for. Specifically, detailed dietary histories, including quantitative assessment of daily protein intake, were not systematically recorded in the MMC database, representing an inherent constraint of this retrospective analysis. While standardized dietary counseling provided within the MMC network likely reduced inter-individual variability in macronutrient consumption, we cannot exclude the possibility that unmeasured differences in protein intake contributed to BUN variability. Third, despite extensive adjustment, residual confounding due to unmeasured or imperfectly measured factors (e.g., detailed dietary patterns, physical activity levels, specific medications) cannot be entirely ruled out. Fourth, the diagnosis of HT was based solely on serological criteria (autoantibodies) without histological confirmation, and we lacked data on thyroid ultrasound findings. Future prospective cohort studies with repeated measurements of BUN and thyroid parameters, as well as interventional studies, are warranted to confirm the temporal relationship and explore potential causal mechanisms.

## 5 Conclusions

In conclusion, our study provides evidence that higher BUN levels, particularly within a lower-to-moderate range, are independently and nonlinearly associated with an increased prevalence of HT in patients with T2DM. These findings highlight BUN not only as a renal marker but also as a potential indicator of metabolic-immune interplay, suggesting its utility in risk stratification for autoimmune thyroiditis in this high-risk population. Further research is needed to elucidate the underlying biological mechanisms and to determine whether modulating BUN-related pathways could influence autoimmune risk.

## Data Availability

The raw data supporting the conclusions of this article will be made available by the authors, without undue reservation.
