# Supplementary material for: Association between blood urea nitrogen and the prevalence of Hashimoto's thyroiditis in adults with type 2 diabetes mellitus: a cross-sectional study
Source: Front Nutr. 2026 Feb 20;13:1769524. doi: 10.3389/fnut.2026.1769524 (PMC12962885; doi:10.3389/fnut.2026.1769524)
Supplement: Supplementary file 1 [file Table_1.DOCX]

**Supplementary Table 1 Missing data and miss rates of covariates**

| **Variables** | **Nonmissing** | **Missing** | **Missing Rate (%)** |
| --- | --- | --- | --- |
| Sex | 2054 | 0 | 0.0 |
| Age | 2054 | 0 | 0.0 |
| Education level | 2054 | 0 | 0.0 |
| DBP | 2054 | 1 | 0.0 |
| SBP | 2054 | 1 | 0.0 |
| BMI | 2054 | 0 | 0.0 |
| Duration of diabetes | 2054 | 1 | 0.0 |
| Hypertension | 2054 | 0 | 0.0 |
| Hyperlipidemia | 2054 | 0 | 0.0 |
| Smoking | 2054 | 0 | 0.0 |
| Drinking | 2054 | 0 | 0.0 |
| FBG | 2054 | 1 | 0.0 |
| FCp | 2054 | 25 | 1.2 |
| HbA1c | 2054 | 6 | 0.3 |
| BUN | 2054 | 0 | 0.0 |
| Scr | 2054 | 0 | 0.0 |
| e-GFR | 2054 | 0 | 0.0 |
| UA | 2054 | 2 | 0.1 |
| TG | 2054 | 2 | 0.1 |
| TC | 2054 | 2 | 0.1 |
| HDL-C | 2054 | 7 | 0.3 |
| LDL-C | 2054 | 6 | 0.3 |
| FT3 | 2054 | 64 | 3.1 |
| FT4 | 2054 | 64 | 3.1 |
| TSH | 2054 | 43 | 2.1 |

All abbreviations are listed in Table 1.

**Supplementary Table 2 Logistic regression analyses of HT**

| Variable | OR (95CI) | P value |
| --- | --- | --- |
| Male, n (%) | 1 (0.8~1.25) | 0.998 |
| Age, y | 1 (0.99~1.01) | 0.825 |
| High school education and above, n (%) | 0.72 (0.54~0.96) | 0.024 |
| DBP, mmHg | 0.99 (0.98~1) | 0.014 |
| SBP, mmHg | 1 (0.99~1) | 0.203 |
| BMI, kg/m2 | 0.99 (0.96~1.02) | 0.5 |
| Duration of diabetes, y | 1 (0.99~1.02) | 0.825 |
| Hypertension, n (%) | 1.04 (0.84~1.29) | 0.718 |
| Hyperlipidemia, n (%) | 0.89 (0.7~1.12) | 0.316 |
| Smoking, n (%) | 1.11 (0.87~1.42) | 0.414 |
| Drinking, n (%) | 0.81 (0.59~1.12) | 0.209 |
| FBG, mmol/L | 0.98 (0.95~1.01) | 0.131 |
| FCp, ng/mL | 1.04 (0.96~1.11) | 0.342 |
| HbA1c, % | 0.96 (0.92~1) | 0.079 |
| BUN, mmol/L | 1.06 (1.01~1.12) | 0.02 |
| T1 | Ref |  |
| T2 | 1.7 (1.29~2.23) | <0.001 |
| T3 | 1.56 (1.18~2.06) | 0.002 |
| Scr, μmol/L | 1 (1~1.01) | 0.188 |
| e-GFR, mL/min per 1.73 m2 | 1 (0.99~1) | 0.035 |
| UA, μmol/L | 1 (1~1) | 0.297 |
| TG, mmol/L | 0.98 (0.93~1.03) | 0.371 |
| TC, mmol/L | 1.01 (0.93~1.1) | 0.792 |
| HDL-C, mmol/L | 0.84 (0.59~1.19) | 0.32 |
| LDL-C, mmol/L | 1 (0.9~1.11) | 0.928 |
| FT3, pg/ml | 0.86 (0.73~1.01) | 0.059 |
| FT4, ng/dl | 0.6 (0.39~0.92) | 0.02 |
| TSH, μIU/ml | 0.96 (0.89~1.04) | 0.323 |
| TRAb,(IU/L | 1.01 (1.01~1.01) | <0.001 |
| TPOAb, IU/mL | 1 (1~1) | <0.001 |

All abbreviations are listed in Table 1.

**Supplementary Table 3 Association between BUN and HT in patients with T2DM**

| Variable | Crude | | Model 1 | | Model 2 | | Model 3 | |
| --- | --- | --- | --- | --- | --- | --- | --- | --- |
|  | OR (95%CI) | P value | OR (95%CI) | P value | OR (95%CI) | P value | OR (95%CI) | P value |
| BUN, mmol/L | 0.98 (0.97~0.98) | <0.001 | 0.98 (0.98~0.99) | <0.001 | 0.99 (0.98~0.99) | <0.001 | 0.99 (0.98~0.99) | <0.001 |
| BUN tertiles |  |  |  |  |  |  |  |  |
| T1 | 1(Ref) |  | 1(Ref) |  | 1(Ref) |  | 1(Ref) |  |
| T2 | 0.58 (0.48~0.69) | <0.001 | 0.61 (0.51~0.74) | <0.001 | 0.65 (0.53~0.79) | <0.001 | 0.68 (0.55~0.84) | <0.001 |
| T3 | 0.5 (0.42~0.6) | <0.001 | 0.57 (0.47~0.69) | <0.001 | 0.69 (0.56~0.85) | <0.001 | 0.77 (0.62~0.95) | 0.016 |

Crude: no adjustment; Model 1: adjusted for sex and age; Model 2: adjusted for model 1 + education level, duration of diabetes, BMI, and HbA1c; Model 3: adjusted for model 2 + smoking, drinking, hypertension, hyperlipidemia.
